# Supplementary material for: Mapping the evolution of fertility support policies in China: A content and instrumental analysis
Source: PLoS One. 2025 Oct 9;20(10):e0332137. doi: 10.1371/journal.pone.0332137 (PMC12510515; doi:10.1371/journal.pone.0332137)
Supplement: S1 Appendix — (ZIP) [file pone.0332137.s001.zip › S1 Appendix. 226 original policy documents/224-财政部、人口计生委关于完善人口和计划生育投入保障机制的意见(FBM-CLI-4-162960).doc]

财政部、人口计生委关于完善人口和计划生育投入保障机制的意见

**发布部门：** [财政部](https://www.pkulaw.com/law/chl?Aggs.IssueDepartment=60210) [国家人口和计划生育委员会(含国家计划生育委员会)(已撤销)](https://www.pkulaw.com/law/chl?Aggs.IssueDepartment=60304) [机构沿革](javascript:void(0))

**发文字号：**财教〔2011〕558号

**发布日期：**2011.11.16

**实施日期：**2011.11.16

**时效性：** [失效](https://www.pkulaw.com/law/chl?Aggs.TimelinessDic=02)

**效力级别：** [部门工作文件](https://www.pkulaw.com/law/chl?Aggs.EffectivenessDic=XE0303)

**法规类别：** [人口计划管理](https://www.pkulaw.com/law/chl?Aggs.Category=09701) [计划生育统计财会](https://www.pkulaw.com/law/chl?Aggs.Category=09703)

**失效依据：**[财政部、卫生计生委关于印发《计划生育服务补助资金管理暂行办法》的通知[失效]、](javascript:TMP(269182,0,'chl'))[财政部关于公布废止和失效的财政规章和规范性文件目录](javascript:TMP(339305,0,'chl'))(第十三批)的决定

财政部、人口计生委关于完善人口和计划生育投入保障机制的意见
（财教[2011]558号）

各省、自治区、直辖市、计划单列市财政厅（局）、人口计生委，新疆生产建设兵团财务局、人口计生委：
　　为贯彻落实党中央、国务院关于全面加强人口和计划生育工作的决策部署，进一步强化公共财政对人口和计划生育的保障责任，支持做好人口和计划生育工作，促进人口长期均衡发展，经国务院同意，现就完善人口和计划生育投入保障机制提出以下意见：
　　**一、**充分认识完善人口和计划生育投入保障机制的重要意义
　　计划生育是我国必须长期坚持的一项基本国策。人口和计划生育工作是政府履行社会管理和公共服务职能的重要组成部分。党中央、国务院历来高度重视人口和计划生育事业，《[中共中央国务院关于全面加强人口和计划生育工作统筹解决人口问题的决定](https://www.pkulaw.com/chl/5e7b01010a4102ffbdfb.html#class=alink)》（中发〔2006〕22号）明确了稳定低生育水平、统筹解决人口问题的目标和任务，就完善人口和计划生育工作的相关社会经济政策以及保障机制提出了明确要求。各地区、各有关部门认真贯彻落实中发［2006］22号文件精神，切实加大财政投入力度，做到“人口和计划生育财政投入增长幅度要高于经常性财政收入增长幅度”，按期实现了到2010年末“各级财政投入人口和计划生育事业费达到人均30元”的投入目标，促进了人口和计划生育事业稳定健康发展。
　　但从长远发展来看，人口和计划生育财政投入仍存在经费投入总量不足，财政分担机制不尽合理，奖励优惠政策落实不到位，基层财政负担过重等突出问题。当前和今后一段时期，我国人口发展既存在老问题，又面临新情况，人口总量持续增长，人口结构和素质问题凸显，人口对资源环境和经济社会发展的约束增强，进一步做好人口和计划生育工作，意义十分重大，任务十分艰巨。根据党的十七届五中全会关于“全面做好人口工作”、“坚持计划生育基本国策，逐步完善政策，促进人口长期均衡发展”等要求，必须进一步完善相关政策措施，切实解决财政投入问题，建立健全促进人口和计划生育事业协调发展的投入保障机制。

　　**二、**完善人口和计划生育投入保障机制的基本原则
　　按照建立健全政府间财力与事权相匹配的财政体制改革方向，建立健全“财政为主，稳定增长，分级负担，分类保障，城乡统筹”的人口和计划生育投入保障机制。
　　（一）财政为主。继续完善坚持以公共财政为主的人口和计划生育投入保障机制，切实落实社会抚养费“收支两条线”规定，各地依法征收的社会抚养费按规定缴入地方国库，所需经费纳入地方财政预算予以保障。
　　（二）稳定增长。继续坚持“人口和计划生育财政投入增长幅度要高于经常性财政收入增长幅度”的要求，确保人口和计划生育财政投入逐年稳定增长。
　　（三）分级负担。明确中央与地方人口和计划生育公共管理、服务的支出责任，完善中央与地方人口和计划生育经费分担机制。
　　（四）分类保障。合理划分人口和计划生育经费类别，建立和完善分类别、分区域、按比例的投入保障机制。
　　（五）城乡统筹。对城乡计划生育家庭和育龄群众提供统一、均等化的计划生育奖励和服务。

　　**三、**完善人口和计划生育投入保障机制的主要内容
　　进一步完善投入保障机制，明确投入主体，突出投入重点，细化投入措施，强化投入责任，不断健全人口和计划生育财政投入政策体系，确保人口和计划生育经费稳定增长。
　　（一）切实加大财政投入力度。各级财政部门要把人口和计划生育经费纳入各级政府财政预算，逐年增加投入，确保法律法规规定的各项奖励扶助和优惠政策、基层人口和计划生育技术服务机构、计划生育经常性工作、计划生育免费基本技术服务等经费的落实。
　　（二）合理划分人口和计划生育经费类别，建立和完善经费分级负担机制。根据全国人口和计划生育事业改革与发展任务，将各级财政负担的人口和计划生育经费按支出功能划分为利益导向、公共服务、能力建设和管理运行四大类，具体为：
　　1.利益导向类。主要包括按照法律、法规和政策规定，对计划生育家庭的各项奖励、扶助和保障性支出。
　　国家规定的计划生育利益导向重大项目所需经费，由中央和地方按比例共同负担。其中，中央财政按照对西部地区负担80%、中部地区负担50%的比例安排专项资金，对东部地区给予奖励性补助。逐步完善农村部分计划生育家庭奖励扶助制度、计划生育“少生快富”工程和计划生育家庭特别扶助制度，建立奖励和扶助标准的动态调整机制。
　　加快独生子女父母奖励费制度改革。按照国家和各地区有关规定落实独生子女父母奖励经费。其中：国家公务员的独生子女父母奖励费从财政部门核拨的行政运行经费中列支，事业单位工作人员的独生子女父母奖励费从事业单位经费中列支；企业发放给职工的独生子女父母奖励费由企业负担，在企业福利费中列支，并可按规定予以税前扣除；农村居民以及城镇非从业居民的独生子女父母奖励费纳入地方财政预算予以保障，中央财政对困难地区的农村居民独生子女父母奖励费给予适当补助。实行城镇独生子女父母年老奖励制度的地区，由地方财政负责落实奖励经费。
　　2.公共服务类。主要包括按照法律、法规和政策规定，对常住人口（含流动人口）的计划生育、生殖健康、优生优育、免费技术服务、宣传教育等支出。
　　国家免费提供的计划生育避孕药具，继续由中央财政全额负担，并依据育龄人群实际需求核定经费。国家免费孕前优生健康检查经费，中央财政按照对西部地区负担80%、中部地区负担50%、东部地区负担20%的比例给予补助。
　　农村地区计划生育免费技术服务经费由地方财政纳入预算予以保障，具体分担比例由各省（区、市）根据财力状况和技术服务工作量等因素统筹确定，中央财政对西部困难地区给予适当补助。城镇非从业居民的计划生育手术费用，由地方财政部门安排专项经费，在人口和计划生育事务经费中列支。城镇职工的计划生育手术费用按现有渠道解决。
　　3.能力建设类。主要包括人口和计划生育服务设施修缮，流动服务车和设备运行维护，信息化建设，改善人口和计划生育基础条件，队伍建设与人员培训等支出。
　　根据基层计划生育服务机构的功能和实际需要，按照优化资源配置的原则和规定的标准，研究制定技术服务、信息、宣传、培训等业务装备规划和年度实施计划，所需经费由地方财政负担，中央财政给予适当补助。
　　4.管理运行类。主要包括各级人口和计划生育管理服务部门的人员经费、公用经费，村（社区）聘用计生人员补贴，人口发展战略研究，人口出生性别比综合治理，人口和计划生育目标责任制考核及实施重大项目的管理运行等支出，由本级财政予以保障。
　　（三）鼓励、引导社会资金投入，建立多渠道筹资体制。国家鼓励企业、社会团体和个人投入人口和计划生育事业，鼓励按照国家有关规定设立人口和计划生育公益基金。积极运用项目合作机制，吸引国内外资金。

　　**四、**工作要求
　　完善人口和计划生育投入保障机制工作，涉及面广，政策性强。各级财政和人口计生部门要从全局出发，充分认识完善人口和计划生育投入保障机制的重要意义，周密部署，统筹安排，扎扎实实把各项要求贯彻落实到位。
　　（一）加强组织领导，搞好协调配合。各级财政和人口计生部门要按照中央关于“责任到位、措施到位、投入到位、落实到位”的要求，切实加强对完善人口和计划生育投入保障机制工作的组织领导，加强协调，密切配合，按照本意见的要求，抓紧制定切实可行的实施方案，进一步完善相关管理办法。财政部、人口计生委要加强对完善人口和计划生育投入保障机制工作的指导和协调。
　　（二）落实分担责任，确保投入到位。省级财政部门要会同人口计生部门研究制定本地区各级财政的经费分担办法，进一步加大对下转移支付力度，统筹考虑与相关部门政策和制度的有效衔接，有效利用医疗卫生资源，增强基层计划生育部门提供公共服务的能力，促进基本公共服务均等化。
　　建立和完善县级人口和计划生育基本财力保障机制，保障县级人口计生部门履行管理和服务职能所必须的人员经费、公用经费以及法律法规规定的各项奖励、扶助和优惠政策、计划生育免费技术服务等民生支出。
　　（三）完善考核办法，强化资金管理。各地区要将财政投入纳入人口和计划生育工作目标管理责任制考核，明确责任。人口计生部门要切实加强预算管理，健全各项资金支付管理制度，严格按照预算办理各项支出，确保资金分配及时、使用规范、安全和有效；要进一步加强预算编制、会计核算等财务基础工作，全面推进财政资金科学化、精细化管理，努力提高资金使用效益。财政部和人口计生委将加强监督检查，对工作实绩好的地方，在安排中央有关专项转移支付资金时给予奖励。

财政部
人口计生委
二○一一年十一月十六日

© 北大法宝：（[www.pkulaw.com](https://www.pkulaw.com/)) 专业提供法律信息、法学知识和法律软件领域各类解决方案。北大法宝为您提供丰富的参考资料，正式引用法规条文时请与标准文本核对。
欢迎查看所有[产品和服务](https://www.pkulaw.com/helps/2.html)。法宝快讯：[如何快速找到您需要的检索结果？](https://www.pkulaw.com/helps/70.html)[法宝](https://www.pkulaw.com/helps/69.html)V6有何新特色？

扫描二维码阅读原文

**原文链接：**<https://www.pkulaw.com/chl/7dedc9a68c27375dbdfb.html>
